# Abrasive Waterjet Machining of r-GO Infused Mg Fiber Metal Laminates: ANFIS Modelling and Optimization Through Antlion Optimizer Algorithm

**DOI:** 10.3390/ma18194480

**Published:** 2025-09-25

**Authors:** Devaraj Rajamani, Mahalingam Siva Kumar, Arulvalavan Tamilarasan

**Affiliations:** 1 Centre for Advanced Materials Processing, Department of Mechanical Engineering, Vel Tech Rangarajan Dr. Sagunthala R&D Institute of Science and Technology, Chennai 600062, India; 2 Department of Mechanical Engineering, SRM TRP Engineering College, Tiruchirappalli 621105, India; lawan.sisa@gmail.com; 3 Department of Artificial Intelligence and Data Science, Saveetha School of Engineering, Saveetha Institute of Medical and Technical Sciences (SIMATS), Chennai 602105, India; tamilarasana.sse@saveetha.com

**Keywords:** fiber metal laminates, abrasive waterjet machining, intelligent modeling, ANFIS, optimization, antlion algorithm

## Abstract

This research proposes an intelligent modeling and optimization strategy for abrasive waterjet machining (AWJM) of magnesium-based fiber metal laminates (FMLs) reinforced with reduced graphene oxide (r-GO). Experiments were designed using the Box–Behnken method, considering waterjet pressure, stand-off distance, traverse speed, and r-GO content as inputs, while kerf taper (*Kt*), surface roughness (*Ra*), and material removal rate (*MRR*) were evaluated as outputs. Adaptive Neuro-Fuzzy Inference System (ANFIS) models were developed for each response, with their critical optimized hyperparameters such as cluster radius, quash factor, and training data split through the dragonfly optimization (DFO) algorithm. The optimized ANFIS networks yielded a high predictive accuracy, with low RMSE and MAPE values and close agreement between predicted and measured results. Four metaheuristic algorithms including particle swarm optimization (PSO), salp swarm optimization (SSO), whale optimization algorithm (WOA), and the antlion optimizer (ALO) were applied for simultaneous optimization, using a TOPSIS-based single-objective formulation. ALO outperformed the others, identifying 325 MPa waterjet pressure, 2.5 mm stand-off, 800 mm/min traverse speed, and 0.00602 wt% r-GO addition in FMLs as optimal conditions. These settings produced a kerf taper of 2.595°, surface roughness of 8.9897 µm, and material removal rate of 138.13 g/min. The proposed ANFIS-ALO framework demonstrates strong potential for achieving precision and productivity in AWJM of hybrid laminates.

## 1. Introduction

Fiber metal laminates (FMLs) are a class of advanced hybrid materials engineered by combining thin metal sheets with fiber-reinforced composite layers, effectively merging the benefits of both constituents. This synergistic design imparts a high strength-to-weight ratio, improved fatigue and impact resistance, and enhanced corrosion and thermal stability, making them highly attractive for aerospace, automotive, marine, and defense applications [1,2]. Magnesium-based FMLs, in particular, offer additional weight savings compared to aluminum or titanium-based systems while maintaining competitive mechanical performance [3,4]. The integration of nano-fillers such as reduced graphene oxide (r-GO) into the polymer matrix has recently emerged as a strategy to further improve interlaminar bonding, damage tolerance, and thermal conductivity, thereby expanding the functional capabilities of these materials [5,6].

Despite these advantages, the heterogeneous nature of FMLs poses significant challenges in conventional machining operations. The alternating metal–composite structure results in disparate material removal behavior, often leading to defects such as delamination, burr formation, fiber pull-out, and reduced surface quality [7,8]. Abrasive waterjet machining (AWJM), a cold-cutting, mechanical erosion-based process, has gained prominence as an alternative, offering minimal thermal damage, the ability to cut complex profiles, and suitability for a wide range of materials [9,10]. In AWJM, process performance is strongly influenced by a set of controllable parameters including waterjet pressure, stand-off distance, traverse speed, and abrasive characteristics. Achieving optimal kerf geometry, superior surface finish, and high material removal rates requires careful tuning of these parameters due to the inherently nonlinear and interdependent nature of the process [11,12].

Accurate modeling of AWJM responses is essential for understanding process behavior and guiding parameter optimization. Adaptive Neuro-Fuzzy Inference Systems (ANFIS) have been successfully applied to capture nonlinear relationships in various manufacturing processes by combining the learning capabilities of artificial neural networks with the linguistic reasoning of fuzzy logic [13]. The predictive accuracy of ANFIS models, however, depends heavily on the selection of structural and training parameters such as cluster radius, quash factor, and the proportion of training/testing data [14]. Metaheuristic optimization algorithms have proven effective for tuning these parameters, enabling improved convergence and generalization [15]. In the recent times, numerous researchers have effectively used ANFIS models for correlating the interrelationship between the parameters of advanced manufacturing processes, especially for non-traditional machining processes. Singh et al. [16] used ANFIS to investigate the effects of pulse-on time, pulse-off time, and current on *MRR*, overcut, and taper angle in EDM of super duplex stainless steel; subsequent multi-objective optimization via a hybrid GRA-PCA method yields a 5.92% improvement in *MRR*, a 27.1% reduction in overcut, and a 75% decrease in taper angle. Rajamani et al. [17] adopted an integrated ANFIS and whale optimization algorithm for the intelligent modeling and optimization of solid-state laser cutting process and found it to be an efficient approach for the prediction of optimal laser cutting parameters. Abdullah Eaysin et al. [18] successfully implemented the ANFIS model for predicting the quality characteristics of laser-beam-machined AISI -P20 mold steel. Denic et al. [19] demonstrated the adoptability of an ANFIS model for the prediction of dross formation in a plasma arc cutting process and found that the developed model performed efficiently with subsidized error values. Siva Kumar et al. [20] also utilized an ANFIS model for the prediction of surface roughness and kerf taper of the plasma arc cutting process. They have found that the ANFIS model performed better than the regression model.

Apart from the modeling, optimization of the AWJ process parameters is essential to achieve the best possible cutting quality. Although several conventional statistical optimization techniques are available, they often fall short in handling the complex, multi-objective nature of machining processes [21,22]. In recent times, several researchers have applied metaheuristic algorithms such as PSO, SSO, and WOA for machining process optimization, reporting promising but sometimes inconsistent results due to their tendency towards premature convergence or slow exploitation. PSO, while widely used, may converge prematurely near local optima under complex multimodal landscapes. SSO has shown a good exploration ability but suffers from stagnation in later iterations. WOA offers a competitive performance for continuous optimization but may require more iterations for convergence due to its spiral update mechanism. The antlion optimizer (ALO), in contrast, has demonstrated strong global search capability with adaptive exploration–exploitation balance, making it highly suitable for solving nonlinear, multi-objective manufacturing problems [23]. ALO has recently been used by several researchers to solve complex real-world problems such as instance reduction for data processing, site layout optimization, optimization of switched reluctance motors, and wireless power transfer systems [24,25,26,27]. When integrated with ANFIS, ALO can serve both as a parameter-tuning tool for modeling and as an optimizer for determining process parameter settings that achieve desired multiple performance objectives. This dual use is particularly advantageous in AWJM of advanced composite laminates, where multiple responses such as kerf taper, surface roughness, and material removal rate must be simultaneously optimized.

In this context, the present study aims to develop an ANFIS modeling framework for predicting AWJM performance metrics of r-GO-reinforced Mg-based FMLs, with ANFIS parameters optimized using the dragonfly optimization (DFO) algorithm. The novelty of this work lies in three key aspects: (i) the fabrication and AWJM of Mg-based FMLs reinforced with r-GO, which has not been widely reported in the literature; (ii) the development of a dragonfly optimization (DFO)-tuned ANFIS modeling framework capable of accurately predicting kerf taper, surface roughness, and *MRR* simultaneously; (iii) the comparative evaluation of four metaheuristic algorithms, with ALO emerging as the most effective for multi-response optimization. This integrated modeling–optimization approach provides a robust decision-making tool for achieving precision and productivity in the AWJM of next-generation hybrid laminates.

## 2. Materials and Methods

### 2.1. Experimental Methodology

Response Surface Methodology (RSM) is a powerful set of statistical and mathematical techniques designed to model and analyze problems where several input variables influence a response of interest. This methodology is particularly valuable for optimizing processes, improving performance, and developing new products. Among the various designs used in RSM, Box–Behnken design is highly efficient and rotatable, making it a preferred choice for many experimental setups [28]. Box–Behnken design strategically places experimental points at the midpoints of the edges of the experimental region, excluding the extreme corners, thereby ensuring reliable and practical results with a reduced number of runs. This approach helps in avoiding the impractical and sometimes unsafe extreme conditions, ensuring feasible and efficient experimentation. The mathematical model used in RSM to represent the relationship between input variables and the response is typically a second-order polynomial equation, expressed as

(1) $$Y=C_{0}+\sum_{i=1}^{k}C_{i}x_{i}+\sum_{i}\sum_{j}C_{ij}x_{i}x_{j}+\sum_{i=1}^{k}C_{ii}x_{i}^{2}+\sigma$$

where σ signifies the statistical distribution error, $C_{i}$, $C_{ij}$ and $C_{ii}$ indicate the coefficient terms of the input parameters, Y indicates the output response, and k indicates the number of output variables.

### 2.2. Adaptive Neuro Fuzzy Inference System

The Adaptive Neuro-Fuzzy Inference System (ANFIS) is a hybrid intelligent system that combines the learning capabilities of neural networks with the reasoning capabilities of fuzzy logic. ANFIS leverages the strengths of both approaches to model complex, nonlinear relationships in data, making it a powerful tool for pattern recognition, system identification, and control [29,30]. By integrating fuzzy logic, ANFIS can handle the uncertainties and imprecision associated with real-world problems, while the neural network component provides a mechanism for learning from data and adapting to changing conditions. This combination allows ANFIS to approximate functions and processes effectively, offering a robust framework for a wide range of applications, including engineering, finance, and medicine. The step-by-step implementation of ANFIS is as follows:

*Step 1: Data Collection and Pre-processing*

Collect a dataset consisting of input–output pairs $\left(x_{i},y_{i}\right)$ where i=1,2,…,N. Normalize the input data $x_{i}$ and the output data $y_{i}$ to fall within a specific range, typically [0, 1] or [−1, 1].

*Step 2: Define the Fuzzy Inference System (FIS)*

Construct the initial Fuzzy Inference System. For each input variable $x_{j}$, define the membership functions $\mu_{A_{j}^{k}}\left(x_{j}\right)$, where $A_{j}^{k}$ represents the kth fuzzy set for the jth input variable. A common membership function is the Gaussian function:

(2) $$\mu_{A_{j}^{k}}\left(x_{j}\right)=\exp\left(-\frac{{\left(x_{j}-c_{j}^{k}\right)}^{2}}{2{\left(\sigma_{j}^{k}\right)}^{2}}\right)$$

where $c_{j}^{k}$, $\sigma_{j}^{k}$ are the center and width of the Gaussian function for the kth fuzzy set of the jth input variable.

*Step 3: Initialize the ANFIS Structure*

Initialize the ANFIS structure by combining the FIS with a neural network architecture.

Layer 1 (Input Layer): Each node i in this layer generates membership grades for each input:

(3) $$O_{i}^{1}=\mu_{A_{j}^{i}}\left(x_{j}\right)$$

Layer 2 (Rule Layer): Each node in this layer represents a rule, and the output is the product of the membership grades:

(4) $$O_{l}^{2}=\omega_{l}=\prod_{j=1}^{m}\mu_{A_{j}^{l}}\left(x_{j}\right)$$

Layer 3 (Normalization Layer): Each node in this layer calculates the normalized firing strength:

(5) $$O_{l}^{3}=\bar{\omega }=\frac{\omega_{l}}{\sum_{l=1}^{L}\omega_{l}}$$

Layer 4 (Defuzzification Layer): Each node in this layer computes a contribution to the output based on the rule’s firing strength:

(6) $$O_{l}^{4}=\bar{\omega_{l}}f_{l}=\bar{\omega_{l}}\left(p_{l}x_{1}+q_{1}x_{2}+\ldots +r_{1}\right)$$

where $p_{l}$, $q_{1}$, and r are the consequent parameters.

Layer 5 (Output Layer): The single node in this layer computes the overall output as the sum of all incoming signals:

(7) $$O^{5}=\sum_{l=1}^{L}\bar{\omega_{l}}f_{l}$$

*Step 4: Train the ANFIS Model*

Train the ANFIS model by adjusting the membership function parameters and the consequent parameters to minimize the error between the actual output $y_{i}$ and the ANFIS output $O_{i}^{5}$.

Error Calculation:

(8) $$E=\frac{1}{N}\sum_{i=1}^{N}{\left(y_{i}-O_{i}^{5}\right)}^{2}$$

Parameter Update: Use a hybrid learning algorithm that combines gradient descent and least squares estimation (LSE). The gradient descent method updates the membership function parameters, while LSE updates the consequent parameters.

*Step 5: Validate the Model*

Validate the trained ANFIS model using a separate testing dataset. Calculate performance metrics such as the Mean Squared Error (MSE):

(9) $$MSE=\frac{1}{N_{test}}\sum_{i=1}^{N_{test}}{\left(y_{test,i}-O_{test,i}^{5}\right)}^{2}$$

*Step 6: Refine the Model*

If the model performance is not satisfactory, refine the ANFIS model by adjusting the number and type of membership functions, re-defining fuzzy rules, or including more data for training.

*Step 7: Implement and Interpret the Results*

Implement the final ANFIS model for the intended application. Interpret the results by analyzing the fuzzy rules and membership functions to gain insights into the relationships within the data.

### 2.3. The Antlion Optimization Algorithm

**Figure 1:**
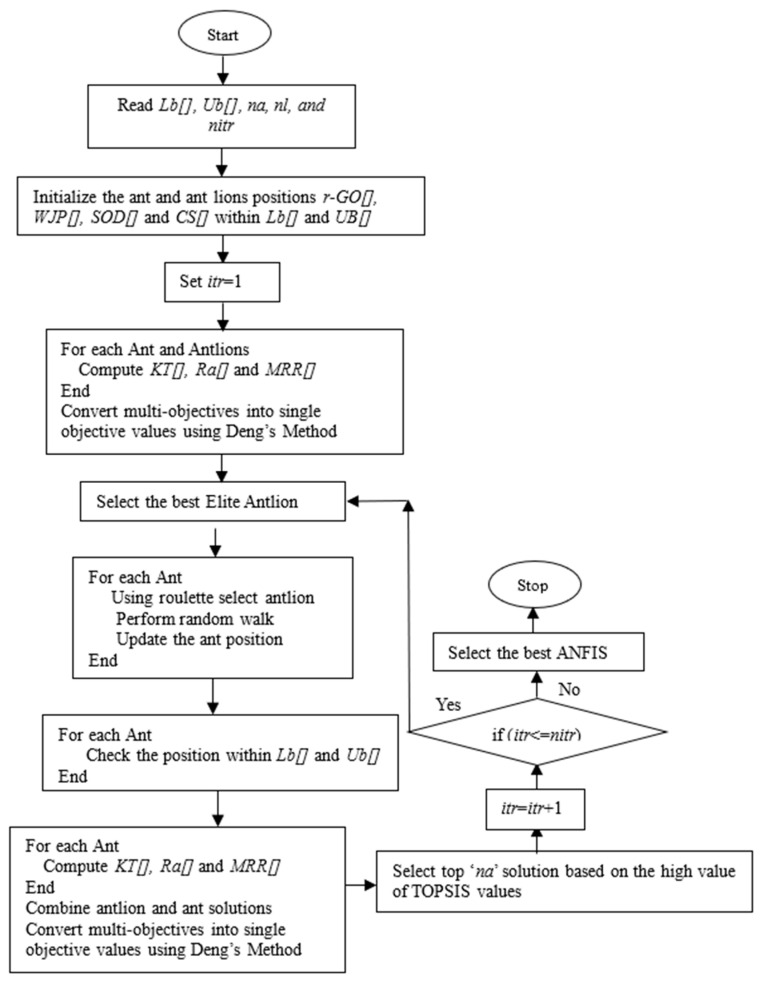
Flowchart for implementation of ALO algorithms to solve multi-objective AWJM problem.

The antlion optimization (ALO) algorithm is a population-based metaheuristic algorithm inspired by the hunting mechanism of antlions in nature, introduced by Mirjalili in 2015 [31]. This algorithm simulates the interaction between ants and antlions in a two-dimensional search space, where antlions represent elite solutions and ants represent potential candidates exploring the solution space. The algorithm relies on random walks of ants influenced by antlions’ traps, mimicking the natural hunting strategy where antlions dig cone-shaped pits to trap ants. ALO effectively balances exploration and exploitation by utilizing both random walks and adaptive selection mechanisms. It has been successfully applied in solving a wide range of real-world optimization problems, especially those with high complexity and nonlinearity. The step-by-step procedure for ALO implementation is described in the flowchart (Figure 1), with its operators as follows.

**Initialization of antlion parameters**

The ALO algorithm simulates the natural interaction between antlions and ants within a trap. In this model, ants explore the search space, while antlions attempt to capture them and improve their own positions through trapping strategies. Because ants exhibit random, unpredictable movements in nature when foraging, their motion is represented mathematically using a random walk, expressed as

(10) $$X\left(t\right)=\left[0,\;cumsum\left(2r\left(t_{1}\right)-1\right),\;cumsum\left(2r\left(t_{2}\right)-1\right),\ldots \ldots ,\;cumsum\left(2r\left(t_{n}\right)-1\right)\right]$$

where cumsum indicates the cumulative sum and the stochastic function $r\left(t\right)$ is defined as follows:

(11) $$r\left(t\right)=\left\{\begin{array}{l} 1\;if\;rand\;>\;0.5\\ 0\;if\;rand\;\leq \;0.5 \end{array}\right\}$$

where rand indicates a random number and lies between 0 and 1, and t denotes the step of random walk. During the initialization of the optimization, the position $\left(M_{a}\right)$ of the ants is defined by the following matrix form:

(12) $$M_{a}=\left(\begin{array}{cccc} a_{1,1} & a_{1,2} & \ldots \ldots & a_{1,d}\\ a_{2,1} & a_{2,2} & \ldots \ldots & a_{2,d}\\ \;: & \;: & \;: & \;:\\ a_{n,1} & a_{n,2} & \ldots \ldots & a_{n,d} \end{array}\right)$$

For the evaluation of each ant, a fitness value $\left(F_{t}\right)$ is assigned for each ant and it can be denoted as

(13) $$M_{oa}=\left[\begin{array}{l} F_{t}\left(\left[a_{1,1},\;a_{1,2},\;\ldots \;a_{1,d}\right]\right)\\ F_{t}\left(\left[a_{2,1},\;a_{2,2},\;\ldots \;a_{2,d}\right]\right)\\ \;:\\ F_{t}\left(\left[a_{n,1},\;a_{n,2},\;\ldots \;a_{n,d}\right]\right) \end{array}\right]$$

In practical terms, the ants are assumed to be hiding somewhere in the search space and saving their positions with fitness value is important for optimization. The following matrices are used to denote their positions ($M_{al}$) and fitness values ($M_{oal}$).

(14) $$M_{al}=\left[\begin{array}{llll} al_{1,1} & al_{1,2} & \ldots \ldots & al_{1,d}\\ al_{2,1} & al_{2,2} & \ldots \ldots & al_{2,d}\\ \;: & \; & \;: & \;:\\ al_{n,1} & al_{n,2} & \ldots \ldots & al_{n,d} \end{array}\right]$$

(15) $$M_{oal}=\left[\begin{array}{l} F_{t}\left(\left[al_{1,1},\;al_{1,2},\;\ldots \;al_{1,d}\right]\right)\\ F_{t}\left(\left[al_{2,1},\;al_{2,2},\;\ldots \;al_{2,d}\right]\right)\\ \;:\\ F_{t}\left(\left[al_{n,1},\;al_{n,2},\;\ldots \;al_{n,d}\right]\right) \end{array}\right]$$

**Random walks of ants**

Ants update their positions within the search space by randomly walking at every step of optimization using Equation (10) through the following normalization:

(16) $$x_{i}^{t}=\frac{\left(x_{i}^{t}-a_{i}\right)\times \left(d_{i}-c_{i}^{t}\right)}{\left(d_{i}^{t}-a_{i}\right)}+c_{i}$$

where $a_{i}\;and\;b_{i}$ are the minimum and maximum random walk of the *i*th variable.

**Trapping in antlions’ pits**

The ants are randomly walking in the search space and they can be trapped by the antlions. This can be mathematically expressed as

(17) $$c_{i}^{t}=al_{j}^{t}+c^{t}$$

(18) $$d_{i}^{t}=al_{j}^{t}+d^{t}$$

**Building the trap**

To simulate the hunting ability of antlions, the algorithm incorporates a roulette wheel selection strategy. In the ALO approach, this operator is used to choose antlions according to their fitness values during the optimization process. This selection method increases the likelihood of stronger antlions capturing ants.

**Sliding the ants towards the antlion**

Using the mechanisms described earlier, antlions can construct traps whose size is proportional to their fitness, while ants move in a random manner. When an antlion detects an ant inside its trap, it throws sand outward from the pit’s center, causing the ant to slide back down while attempting to escape. To mathematically represent this behavior, the radius of the hypersphere defining the ants’ random walk is adaptively reduced. The following equations describe this process:

(19) $$c^{t}=\frac{c^{t}}{I}$$

(20) $$d^{t}=\frac{d^{t}}{I}$$

where I is a ratio, and $c^{t}$ and $d^{t}$ are the vectors of minimum and maximum of all variables at the $t^{th}$ iteration.

**Catching prey and rebuilding the pit**

The hunting process concludes when an ant reaches the pit’s bottom and is seized by the antlion’s jaws. At this point, the antlion drags the prey beneath the sand and consumes it. To replicate this behavior in the algorithm, it is considered that prey capture happens when an ant achieves a better fitness value (moves deeper into the sand) than its corresponding antlion. The antlion then updates its position to match the most recent location of the captured ant, thereby increasing its chances of catching additional prey. The process is mathematically represented by the following equation:

(21) $$al_{j}^{t}=al_{j}^{t}\;if\;f\left(a_{i}^{t}\right)>f\left(al_{j}^{t}\right)$$

**Elitism**

Elitism is a key feature of evolutionary algorithms that ensures the preservation of the best solution(s) identified at any point in the optimization process. In this work, the top-performing antlion in each iteration is recorded and designated as the elite. Being the most fit, the elite is assumed to influence the movement of all ants throughout the iterations. Consequently, it is considered that each ant performs a random walk influenced simultaneously by an antlion selected via the roulette wheel and the elite, as described below:

(22) $$a_{i}^{t}=\;\frac{r_{a}^{t}+r_{e}^{t}}{2}$$

where $r_{a}^{t}$ and $r_{e}^{t}$ indicate the random walk around the antlion selected through roulette wheel and random walk around the elite at the $t^{th}$ iteration, respectively.

### 2.4. AWJ Cutting Experiments of Mg FMLs

**Figure 2:**
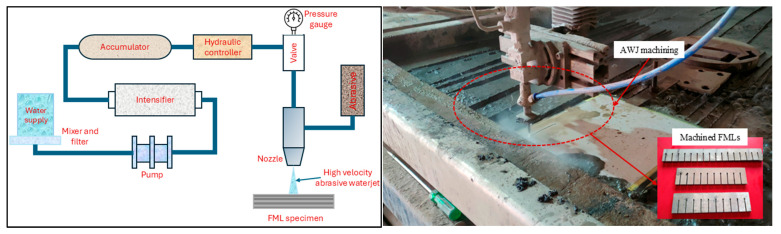
Abrasive waterjet machining schematic and experimental setup and machine FMLs.

Reduced graphene oxide (r-GO)-reinforced Mg-based fiber metal laminates (FMLs) were fabricated using a vacuum-assisted resin infusion technique with an alternating layup of carbon and Kevlar fibers between outer magnesium sheets. The AWJ machining experiments were conducted on three FML variants with 0%, 0.5%, and 1% r-GO filler by weight. A gantry-type CNC abrasive waterjet cutting system (Model G3020, Aquacut Machine Tool, MicroStep, spol. s r.o. Bratislava, Slovakia) was employed, featuring a 0.76 mm carbide nozzle, 350 MPa maximum pressure, and a cutting speed capacity of 15 m/min. Consistent process parameters were maintained for abrasive flow rate (125 g/min), particle size (177 µm), and nozzle angle (90°) throughout the experimental studies. The experimental setup for performing the AWJ cutting studies is shown in Figure 2. Based on the Box–Behnken design, thirty straight slots of 60 mm were machined across the FML samples, considering waterjet pressure (275–325 MPa), stand-off distance (2.5–3.5 mm), cutting speed (600–800 mm/min), and filler content (0–1 wt%) as the key input factors with three levels. Kerf taper (*Kt*), surface roughness (*Ra*), and material removal rate (*MRR*) were quantified using a VMM 4030 video measurement system, white-light interferometer, and high-precision weight balance with a readability of 0.002 g, respectively. The measured response characteristics are listed in Table 1.

## 3. Result and Discussions

### 3.1. Statistical Analysis of Regression Models

**Figure 3:**
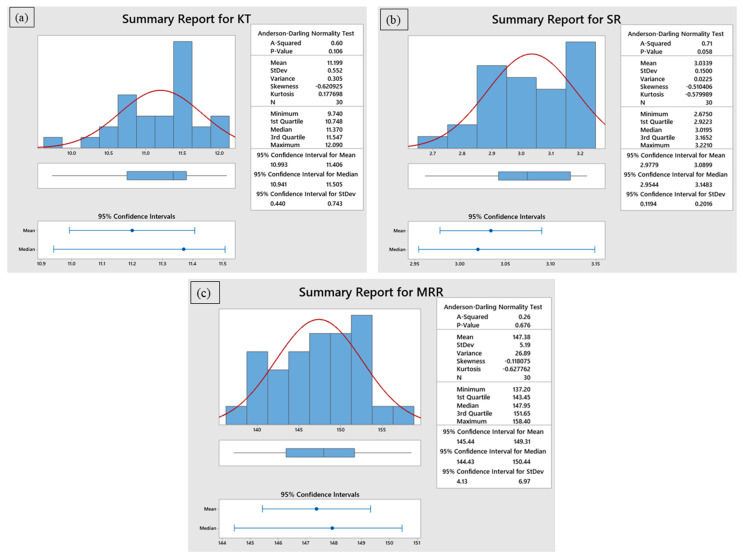
Anderson–Darling normal probability analysis for (**a**) *Kt*, (**b**) *Ra*, and (**c**) *MRR*.

The influence of process parameters on *Kt*, *Ra*, and *MRR* was examined through analysis of variance (ANOVA), with model adequacy further validated using the Anderson–Darling (A–D) normality test. The results are summarized in Table 2, and the residual distributions are illustrated in Figure 3a–c. For *Kt*, the model exhibited high significance with an F-value of 298.81 and a *p*-value less than 0.0001, confirming that the selected factors substantially influence the response. The coefficient of determination (R^2^ = 99.53%) and adjusted R^2^ (99.2%) indicate a strong fit between experimental and predicted values. Among the variables, r-GO content had the greatest influence (F = 627.37), followed by stand-off distance and cutting speed. The lack of fit was statistically insignificant (*p* = 0.4726), confirming that the model fits the data well. The A–D plot for *Kt* (Figure 3a) shows that the residuals follow a normal distribution, validating the regression assumptions. In the case of *Ra*, the model was statistically significant (*p* < 0.0001) with an F-value of 28.32, R^2^ of 95.83%, and adjusted R^2^ of 92.45%. Waterjet pressure emerged as the most significant parameter (F = 38.27), followed by cutting speed and r-GO content. The interaction and curvature effects are also captured effectively, as seen from the significant model terms. The lack of fit was not significant (*p* = 0.2903), suggesting the model’s adequacy. As shown in the A–D plot for *Ra* (Figure 3b), the residuals fall closely along the reference line, confirming normal error distribution. For *MRR*, the regression model showed a high level of significance, with an F-value of 55.77 and *p* < 0.0001. The model demonstrated excellent predictive capability, with R^2^ = 97.15% and adjusted R^2^ = 95.41%. r-GO content again dominated the influence (F = 107.99), followed by waterjet pressure. Although stand-off distance and cutting speed had comparatively lower F-values, their contributions were still statistically meaningful (*p* < 0.05). The A–D plot for *MRR* (Figure 3c) confirmed that the residuals were normally distributed, and the lack-of-fit was found to be insignificant (*p* = 0.2178), indicating the model’s reliability for prediction.

### 3.2. ANFIS Modeling of AWJ Performance Characteristics

The AWJ machining process exhibits strong nonlinearities between input parameters and performance characteristics, making accurate predictive modeling essential. In this study, a machine learning prediction tool named ANFIS was employed, with its key parameters such as cluster radius (RADII), quash factor, and training data proportion considered for prediction and optimized through the dragonfly optimization (DFO) algorithm. This hybrid approach reduces reliance on user-defined trial-and-error parameter selection, instead utilizing swarm intelligence to achieve an optimal configuration that minimizes prediction errors.

The modeling used a subtractive clustering-based Sugeno-type ANFIS structure, where the RADII value controls the influence range of each cluster center. A smaller RADII generally increases model precision by generating more localized membership functions. In this work, RADII values were explored within 0.1–0.5, with quash factors ranging from 2 to 3, and training data proportions between 60% and 85%, as shown in Table 3. The DFO algorithm iteratively tuned these variables until the network exhibited minimal deviation between training and testing errors. The parameters of the DFO algorithm employed for optimizing the hyperparameters of the ANFIS network are presented in Table 4. These values were determined after multiple trial iterations and are regarded as the most suitable training parameters for the current study.

The optimized ANFIS parameter sets obtained for each response characteristic through DFO as mentioned in Table 5 reveal notable variations. For *Kt* prediction, the optimal RADII was 0.4074 with a quash factor of 2.5211 and 78.96% of data used for training. For *Ra*, a higher RADII value of 0.2221 and a lower quash factor of 2.753 were found effective, with approximately 78.56% training data, indicating that *Ra* prediction benefited from a broader cluster influence but required a larger proportion of data for testing to maintain accuracy. *MRR* prediction used the smallest RADII (0.4258) and the highest training data proportion (79.04%), suggesting that more localized membership functions and greater exposure to training samples improved its predictive stability. These distinctions imply that the sensitivity of each performance characteristic to AWJ control parameters is unique, necessitating tailored ANFIS configurations for optimal results.

**Figure 4:**
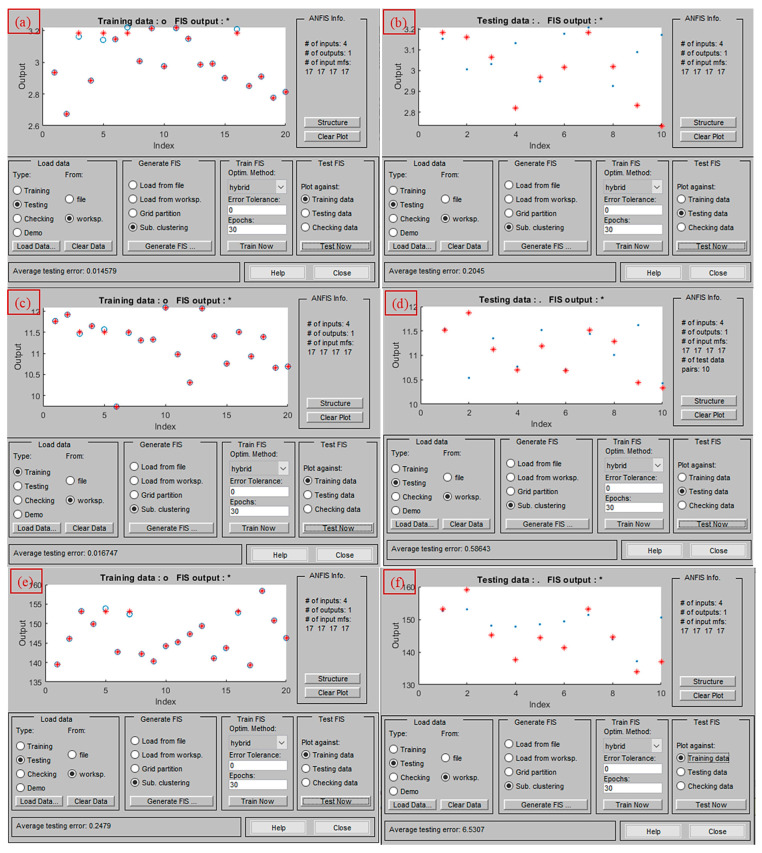
Training and testing error of ANFIS models for (**a**,**b**) kerf taper, (**c**,**d**) surface roughness, and (**e**,**f**) material removal rate.

The training and testing error profiles shown in Figure 4a–f for *Kt*, *Ra*, and *MRR* confirm that the proposed DFO-tuned ANFIS models converged effectively, maintaining low and stable error values during testing. The optimized ANFIS parameter sets mentioned in Table 5 for each response show variation in RADII, quash factor, and training data percentage, reflecting the distinct sensitivity of *Kt*, *Ra*, and *MRR* to the AWJ input parameters. The statistical validation results demonstrate that the developed models achieved a high prediction accuracy, with low RMSE and MAPE values for all responses (Table 5). *Kt* recorded the lowest RMSE (0.0151%), followed by *Ra* (0.2288%), while *MRR* showed slightly higher error levels but remained within acceptable predictive limits.

**Figure 5:**
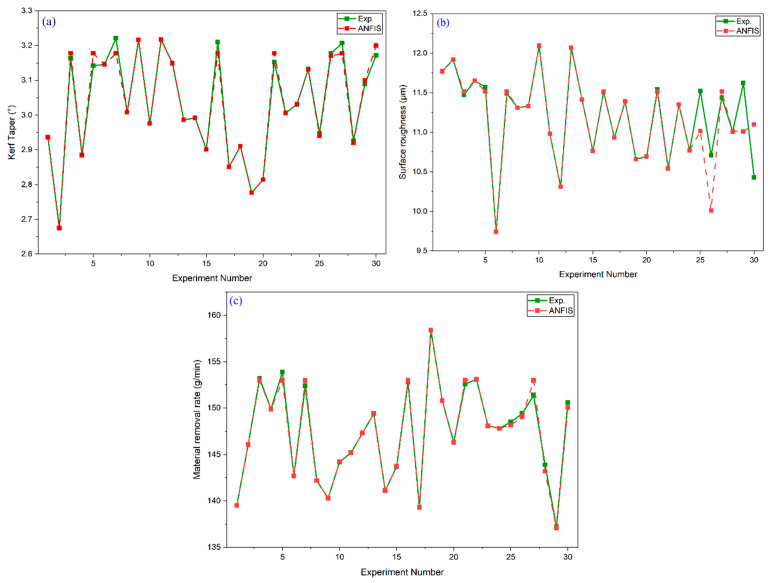
ANFIS predicted vs. experimental response values for (**a**) kerf taper, (**b**) surface roughness, and (**c**) material removal rate.

The predicted versus experimental plots shown in Figure 5a–c display close alignment between model outputs and measured values, with points distributed tightly around the ideal 45° reference line. This consistency across all three responses confirms that the DFO-tuned ANFIS models effectively capture the complex nonlinear behavior of the AWJ process and can reliably estimate process performance based on given input settings. Statistical examination of the residuals indicated that the differences between predicted and experimental values were not significant at the 95% confidence level. Furthermore, model predictions for unseen test data showed only a marginal reduction in accuracy, confirming its suitability for practical deployment in process planning and optimization. Overall, the integration of DFO for optimizing the ANFIS parameters provides a robust framework for predictive modeling of AWJ machining. The approach delivers high-fidelity predictions, strong generalization capability, and minimal prediction error, making it a valuable tool for future multi-response process optimization in AWJ applications.

### 3.3. Influence of AWJ Cutting Parameters on the Response Characteristics

***Influence on Kerf Taper (Kt)***

Kerf taper is a critical quality indicator in abrasive waterjet machining, representing the difference in width between the top and bottom of the cut. A high kerf taper leads to poor dimensional accuracy and may require secondary machining, especially in precision applications. Therefore, minimizing taper is essential for achieving uniform cuts and maintaining the integrity of composite materials.

**Figure 6:**
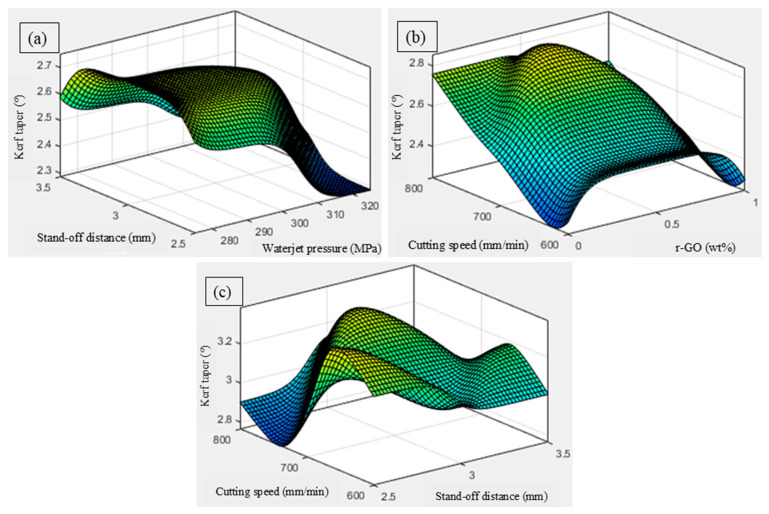
Influence of AWJC parameters on kerf taper: (**a**) stand-off distance vs. waterjet pressure, (**b**) cutting speed vs. wt% of r-GO, (**c**) cutting speed vs. stand-off distance.

The influence of waterjet pressure and stand-off distance on the kerf taper is depicted in Figure 6a. This plot shows that kerf taper decreases at higher pressures (3200 MPa) and lower stand-off distances. At higher pressures, the jet has enough momentum to maintain straight penetration, while minimal stand-off keeps the jet tight and concentrated. As stand-off increases, taper worsens due to divergence and loss of energy [32]. This reflects classical AWJM physics and matches taper-reduction strategies. The 3D surface plot (Figure 6b) indicates the impact of r-GO addition and the cutting speed on the kerf taper. Kerf taper reduces at a high cutting speed and moderate r-GO content (~0.5 wt.%). Higher speed shortens the exposure time, reducing side erosion. Meanwhile, 0.5 wt.% r-GO improves resistance without causing embrittlement. At high r-GO (>1 wt.%), increased stiffness may lead to chipping, slightly increasing taper. This interaction supports the ANOVA finding where both factors were statistically significant. The ANFIS 3D pot (Figure 6c) shows the impact of stand-off distance and cutting speed on the kerf taper. Taper is minimized at low stand-off and higher traverse speed. The focused jet at low stand-off enhances straight cuts, and increased speed limits lateral erosion. At high stand-off, even higher speed cannot compensate for jet divergence [33]. This combination shows the practical interplay between energy focus and motion control.

***Influence on Surface Roughness (Ra)***

Surface roughness (*Ra*) directly affects the functional performance, fatigue life, and aesthetic appeal of machined components. In AWJM of fiber-reinforced or nanoparticle-reinforced laminates, achieving a smooth surface without delamination or fiber pull-out is challenging. Thus, understanding how input parameters influence *Ra* is vital for ensuring surface integrity without post-processing.

**Figure 7:**
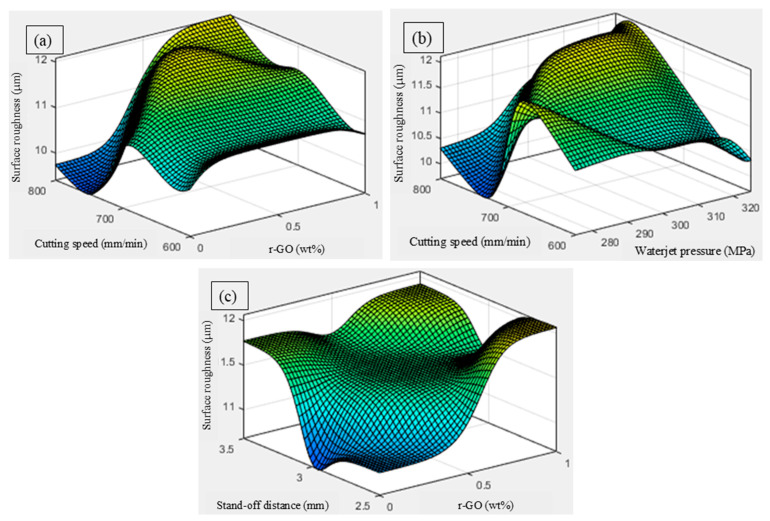
Influence of AWJC parameters on surface roughness: (**a**) cutting speed vs. wt% of r-GO, (**b**) cutting speed vs. waterjet pressure, (**c**) stand-off distance vs. wt% of r-GO.

The surface plot shown in Figure 7a indicates the variation in *Ra* with respect to the inclusion of r-GO and traverse speed. This plot shows that surface roughness is minimized at a moderate r-GO content (0.5 wt.%) and lower traverse speeds (600 mm/min). The inclusion of r-GO enhances matrix hardness and provides better resistance to micro-erosion at the surface. At low traverse speed, the jet has more interaction time per unit length, enabling uniform material removal and smoother surfaces. At higher traverse speeds, insufficient dwell time leads to incomplete erosion, causing surface irregularities [34]. A further increase in r-GO content beyond 0.5 wt.% might increase roughness slightly due to agglomeration and micro-fracturing effects. The surface plot (Figure 7b) illustrates that moderate-to-high waterjet pressure (3000–3300 MPa) combined with lower traverse speeds (600–650 mm/min) leads to an improved surface finish. Higher pressure allows better abrasive particle acceleration, resulting in effective surface shearing and material removal. However, if the speed is too high, the jet cannot erode uniformly, leading to increased surface waviness [35]. This plot reflects the classical balance between jet energy and material exposure time which is an essential interaction in AWJM of reinforced laminates. In the plot (Figure 7c), the surface roughness shows a valley zone at moderate r-GO content (0.5 wt.%) and lower stand-off distance (2–2.5 mm). The proximity of the nozzle ensures a focused jet and stable erosion zone, which when paired with optimal reinforcement, prevents fiber pull-out and surface delamination. At higher stand-off distances, the jet diverges, reducing cutting efficiency and causing more irregular surfaces. Additionally, very high r-GO content increases the risk of brittle fracture and uneven erosion, slightly degrading the surface quality [36].

***Influence on Material Removal Rate (MRR)***

The material removal rate (*MRR*) reflects the productivity and economic efficiency of the AWJM process. A higher *MRR* implies faster machining but must be balanced with quality considerations. For advanced composites, optimizing *MRR* while preserving part integrity is essential for industrial scalability and cost-effective manufacturing.

**Figure 8:**
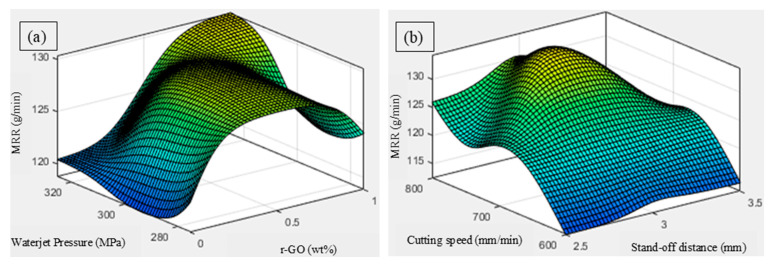
Influence of AWJC parameters on the *MRR*: (**a**) r-GO vs. waterjet pressure, and (**b**) cutting speed vs. stand-off distance.

The three-dimensional ANFIS plot (Figure 8a) illustrates that MRR increases with rising pressure and lower r-GO content. High pressure enhances particle velocity and impact energy, improving penetration and material removal. However, as r-GO increases, *MRR* declines due to higher matrix hardness and reduced erosion susceptibility. This trade-off is typical in reinforced polymer or metal composites, where added stiffness suppresses ductile erosion [37]. This trend aligns well with ANOVA, where r-GO content was the most influential parameter. The 3D surface plot shown in Figure 8b indicates that *MRR* improves with increasing cutting speed and decreasing stand-off distance. Higher speeds promote faster material shearing and debris evacuation, while lower stand-off concentrates energy for efficient material break-up. Excessive stand-off reduces effectiveness due to jet spreading [38,39,40]. This plot highlights the balance between speed-driven productivity and geometric control, supporting experimental optimization observations.

### 3.4. Multi-Response Optimization of AWJ Cutting Parameters Through ALO

To simultaneously optimize *Kt*, *Ra*, and *MRR* in abrasive waterjet machining of hybrid laminates, four metaheuristic algorithms including particle swarm optimization (PSO), salp swarm optimization (SSO), whale optimization algorithm (WOA), and the antlion optimizer (ALO) were employed. The multi-objective problem was reformulated into a single-objective function using the TOPSIS method, where *Kt* and *Ra* were treated as minimization goals and *MRR* as a maximization objective. The optimization framework relied on optimized ANFIS models, developed for each response, to serve as high-fidelity surrogate functions. The quality and predictive accuracy of the ANFIS models were previously validated through statistical performance indicators including RMSE and MAPE. These models effectively captured the nonlinear behavior of the machining system, enabling the algorithms to perform solution searches without additional experimentation.

**Figure 9:**
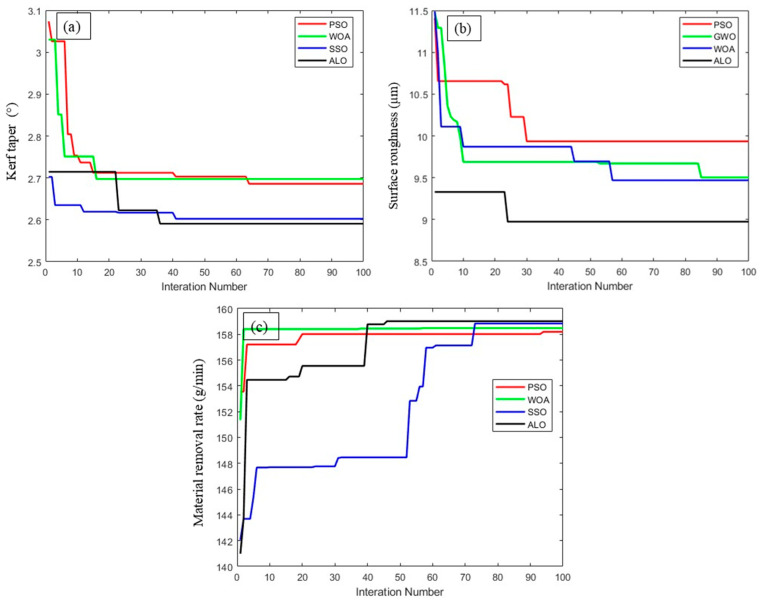
Sample convergence plots of different optimization algorithms: (**a**) kerf taper, (**b**) surface roughness, (**c**) material removal rate.

The control parameters and boundary conditions for all four algorithms are listed in Table 6. Each algorithm was executed independently 17 times, allowing for assessment of solution stability and consistency. The convergence behavior, as observed from the convergence plots (Figure 9a–c), illustrates distinct trends. ALO showed rapid and smooth convergence, demonstrating efficient exploitation after a well-distributed exploratory phase. It consistently reached global optima within fewer iterations and exhibited minimal oscillations. In contrast, PSO showed a moderately stable convergence pattern but with occasional fluctuations, indicative of premature convergence in some trials. WOA maintained a relatively stable performance but converged slowly, likely due to its spiral position update mechanism, which limits exploration in early iterations. SSO demonstrated the most unstable behavior with evident stagnation in later stages, suggesting susceptibility to local optima.

In addition to convergence stability, the effectiveness of each algorithm was further evaluated using performance indices as summarized in Table 7 which is derived from seventeen independent optimization runs. Among the four algorithms, ALO exhibited the highest overall performance index, supported by its lowest standard deviation and best mean fitness value. This confirms its superior ability to consistently arrive at near-global optima across multiple executions. WOA, while marginally behind ALO, also demonstrated competitive performance in terms of stability and average fitness, indicating its suitability for moderately complex optimization landscapes.

The optimization results obtained from these algorithms (Table 8) clearly indicate that ALO outperformed all others, offering the most favorable trade-off among the three responses. It yielded the lowest *Kt* and *Ra*, along with a comparatively high *MRR*, thereby delivering the highest overall performance index. The optimal process conditions obtained using ALO corresponded to a waterjet pressure of 325 MPa, stand-off distance of 2.5 mm, and cutting speed of 800 mm/min with r-GO addition of 0.00602 wt%. These conditions collectively enabled enhanced surface finish, reduced taper, and maximized machining productivity. These results reinforce the finding that ALO’s exploration–exploitation balance, along with its adaptive search mechanism, enabled superior global search behavior, leading to consistently optimized parameter combinations. The comparative analysis of performance indices highlights ALO as the most reliable and efficient algorithm for multi-response optimization of abrasive waterjet machining processes using ANFIS-generated objective models.

The proposed ANFIS–ALO modeling and optimization framework is highly scalable and can be readily adapted for industrial applications where precision cutting of hybrid laminates is required. Potential application sectors include aerospace (fuselage panels, bulkheads), automotive (lightweight structural members), and defense (armor-grade laminates), where dimensional accuracy and surface quality are critical. Since the approach relies on machine learning-based surrogate models, once the model is trained with sufficient experimental data, additional predictions can be made at negligible computational cost, enabling real-time decision support in production environments.

From an economic standpoint, the experimental design employed in this study already minimizes the number of trials required to build accurate predictive models, which reduces development cost. However, full-scale industrial deployment would require validation under higher-pressure AWJM systems and longer production runs to account for nozzle wear, abrasive recycling, and process variability. These aspects present opportunities for future work before large-scale implementation.

## 4. Conclusions

From the experimental investigation, modeling, and optimization of AWJM on r-GO-reinforced Mg-based fiber metal laminates, the key findings are as follows:

- The DFO-assisted ANFIS approach successfully modeled the nonlinear relationships between process parameters and output characteristics, achieving consistently low RMSE and MAPE values with close alignment to experimental data.
- Each performance measure required distinct ANFIS parameter settings: Kt modeling was optimal at a cluster radius of 0.4074, Ra at 0.2221, and MRR at 0.4258, highlighting variation in response sensitivity.
- ANOVA indicated that inclusion of r-GO in the FMLs and waterjet pressure most strongly influenced Kt and MRR, whereas waterjet pressure and traverse speed were dominant for Ra.
- Comparative analysis of metaheuristic optimizers confirmed ALO’s superior convergence behavior, stability, and multi-response performance index. The optimal cutting conditions determined by ALO are 325 MPa waterjet pressure, 2.5 mm stand-off distance, 800 mm/min traverse speed, and 0.00602 wt% r-GO, which yielded minimal kerf taper (2.595°), improved surface quality (8.9897 µm), and a competitive material removal rate (138.13 g/min).
- The hybrid ANFIS–ALO methodology offers a reliable and adaptable framework for process optimization in AWJM, with promising applicability to other composite machining scenarios.
- The results of this study demonstrate that AWJM is a highly suitable process for machining r-GO-infused Mg-based FMLs due to its ability to produce burr-free cuts without thermal damage or delamination. This makes it advantageous for manufacturing high-performance lightweight components in sectors such as aerospace and automotive, where dimensional accuracy and structural integrity are critical.

## Figures and Tables

**Table 1:** AWJ experimental parameters and their measured response values.

Exp. No	Input Parameters				Response Characteristics		
	r-GO (wt%)	Waterjet Pressure (MPa)	Stand-Off Distance (mm)	Cutting Speed (mm/min)	Surface Roughness (µm)	Kerf Taper (°)	Material Removal Rate (g/min)
1	0	275	3	700	10.71	3.177	149.4
2	1	275	3	700	11.52	2.948	148.5
3	0	325	3	700	10.69	2.814	146.3
4	1	325	3	700	11.39	2.911	158.4
5	0.5	300	2.5	600	11.33	3.216	140.3
6	0.5	300	3.5	600	11.31	3.008	142.2
7	0.5	300	2.5	800	10.76	2.901	143.7
8	0.5	300	3.5	800	11.41	2.992	141.1
9	0	300	3	600	11.62	3.089	137.2
10	1	300	3	600	10.77	3.132	147.8
11	0	300	3	800	9.74	3.146	142.7
12	1	300	3	800	12.09	2.976	144.2
13	0.5	275	2.5	700	11.35	3.031	148.1
14	0.5	325	2.5	700	10.66	2.777	150.8
15	0.5	275	3.5	700	11.01	2.926	143.9
16	0.5	325	3.5	700	11.65	2.885	149.9
17	0	300	2.5	700	10.93	2.851	139.3
18	1	300	2.5	700	12.07	2.986	149.4
19	0	300	3.5	700	11.77	2.936	139.5
20	1	300	3.5	700	11.92	2.675	146.1
21	0.5	275	3	600	10.98	3.217	145.2
22	0.5	325	3	600	10.43	3.172	150.6
23	0.5	275	3	800	10.31	3.149	147.3
24	0.5	325	3	800	10.54	3.006	153.1
25	0.5	300	3	700	11.54	3.153	152.6
26	0.5	300	3	700	11.51	3.21	152.8
27	0.5	300	3	700	11.47	3.163	153.2
28	0.5	300	3	700	11.49	3.221	152.4
29	0.5	300	3	700	11.57	3.142	153.9
30	0.5	300	3	700	11.44	3.207	151.4

**Table 2:** Analysis of variance for fitted response models.

Source	Sum of Squares	DF	Mean Square	F	Prob. > F
***Kt***					
Model	8.81	12	0.7339	298.81	<0.0001
*A*—r-GO	1.54	1	1.54	627.37	<0.0001
*B*—Waterjet pressure	0.0225	1	0.0225	9.17	0.0076
*C*—Stand-off distance	0.3234	1	0.3234	131.68	<0.0001
*D*—Cutting speed	0.2107	1	0.2107	85.78	<0.0001
Residual	0.0418	17	0.0025		
Lack of fit	0.0306	12	0.0026	1.15	0.4726
Pure error	0.0111	5	0.0022		
Total	8.85	29			
R^2^	99.53%		Adj. R^2^	99.2%	
***Ra***					
Model	0.6251	13	0.0481	28.32	<0.0001
*A*—r-GO	0.0124	1	0.0124	7.27	0.0159
*B*—Waterjet pressure	0.065	1	0.065	38.27	<0.0001
*C*—Stand-off distance	0.0096	1	0.0096	5.67	0.03
*D*—Cutting speed	0.0367	1	0.0367	21.64	0.0003
Residual	0.0272	16	0.0017		
Lack of fit	0.0214	11	0.0019	1.7	0.2903
Pure error	0.0057	5	0.0011		
Total	0.6522	29			
R^2^	95.83%		Adj. R^2^	92.45%	
***MRR***					
Model	757.47	11	68.86	55.77	<0.0001
*A*—r-GO	133.33	1	133.33	107.99	<0.0001
*B*—Waterjet pressure	59.41	1	59.41	48.12	<0.0001
*C*—Stand-off distance	6.6	1	6.6	5.35	0.0328
*D*—Cutting speed	6.45	1	6.45	5.23	0.0346
Residual	22.22	18	1.23		
Lack of fit	18.74	13	1.44	2.07	0.2178
Pure error	3.49	5	0.6977		
Total	779.69	29			
R^2^	97.15%		Adj. R^2^	95.41%	

**Table 3:** Range of ANFIS parameters used for optimization.

ANFIS Parameters	Representation	Range
RADII	Four input parameters and a response value (either *kt* or *R_a_* or *MRR*)	0.1 to 0.5
Quash factor	Used to multiply with RADII values	2 to 3
% of data for training ANFIS model	Number of experiments	60% to 85%

**Table 4:** Parameters of dragonfly algorithm used for ANFIS tuning.

Parameters	Value/Equation
Number of dragonflies (*nd*)	100
Number of iterations (*nitr*)	100
Inertia wt. (*w*) (*w_max_* = 0.8 and *w_min_* = 0.2)	$w=w_{max}-(w_{max}-w_{min})\frac{itr}{nitr}$
Separation wt.	$sw=0.1-\frac{0.1\;*\;itr}{nitr}$
Alignment wt.	$aw=0.1-\frac{0.1\;*\;itr}{nitr}$
Cohesion wt.	$cw=0.1-\frac{0.1\;*\;itr}{nitr}$
Food factor	ff=2 * rand
Enemy factor	$ef=0.1-\frac{0.1\;*\;itr}{nitr}$
Achieve size	100

**Table 5:** Optimized ANFIS parameters obtained through DFO and their corresponding statistical validation values.

RADII Value					%Testing Data	Quash Factor	T_Error_	C_Error_	RMSE	MAPE
r-GO	WP	SOD	CS	*KT*/*Ra*/*MRR*						
0.2960	0.2627	0.4509	0.3421	0.4074	0.7895	2.5210	0.0145	0.204	0.0151	0.2563
0.3906	0.4137	0.2894	0.1424	0.2221	0.7856	2.7530	0.0167	0.586	0.2288	0.8238
0.2334	0.4921	0.3187	0.4939	0.4258	0.7904	2.2142	0.2479	6.530	0.4064	0.1364

**Table 6:** Algorithm parameters and their values used for executing PSO, SSO, WOA, and ALO.

Algorithm	Algorithm Parameters	Range/Value of Parameters
PSO	Learning factors (*C1* and *C2*)	2 and 2
	Inertia weight (*ω*)	0.6
	Particle size (*N*)	30
	No. of iterations (*nitr*)	100
SSO	C1-coefficient to exploration and exploitation	$2e^{-{\left(\frac{4*it}{nitr}\right)}_{2}}$
	C2 and C3	Random values between 0 and 1
	No. of salps (*N*)	30
	No. of iterations (*nitr*)	100
WOA	Number of whales (i = 1, 2, 3,…nw)	Number of solutions
	Position of whale (Xi.)	Combination of variables within their boundary conditions
	Number of dimensions involved in defining the position of whale (j = 1, 2…nd)	Number of independent variables
	Position of prey (Xp.)	Value of best variables
	Fitness of whale (Fik) (k = 1, 2…no)	Response variable
	Stopping criteria	Number of iterations
ALO	No. of antlions (*na*)	100
	No. of iterations (*nitr*)	100
	Accuracy of exploitation	3 to 6 Based on % of no. of iterations
	Achieve size	100

**Table 7:** Performance metrics of different algorithms.

Performance Metric	Probability	Rank Values			
		PSO	WOA	SSO	ALO
Objective value	0.006983	2.43	2.39	2.53	2.65
CI_*Kt*	3.06 × 10^−7^	3.9	1.825	2.025	2.25
CI_*Ra*	3.52 × 10^−10^	3.325	3.5	1.325	1.85
CI_*MRR*	8.72 × 10^−5^	2.1	3.65	2.1	2.15
CV_*Kt*	2.4 × 10^−12^	1.1	2.925	1.975	4
CV_*Ra*	6.19 × 10^−12^	1.65	1.475	2.875	4
CV_*MRR*	2.43 × 10^−11^	1.9	1.1	3.45	3.55
Computational time	2.54 × 10^−10^	1.35	3.95	2.85	1.85
Diversity		3	2	1	4
Spacing value		4	2	3	1
Overall Performance		0.5875	0.5533	0.5793	0.6205

CI—convergence iteration; CV—minimum convergence value.

**Table 8:** Optimal process parameters and response values obtained through different algorithms.

Algorithms	*r-GO* (wt%)	*WP* (MPa)	*SOD* (mm)	*CS* (mm/min)	*Kt* (°)	*Ra* (µm)	*MRR* (g/min)
PSO	0.01810	322.31	2.516	785.64	2.663	9.3958	139.53
WOA	0.02668	323.70	2.558	796.12	2.693	9.2368	140.05
SSO	0.02806	325	2.5	800	2.610	9.0481	138.69
ALO	0.00602	325	2.5	800	2.595	8.9897	138.13

## Data Availability

The original contributions presented in this study are included in the article. Further inquiries can be directed to the corresponding author.
